# Reconstruction of dental roots for implant planning purposes: a retrospective computational and radiographic assessment of single-implant cases

**DOI:** 10.1007/s11548-023-02996-x

**Published:** 2023-07-31

**Authors:** Leonard Simon Brandenburg, Joachim Georgii, Rainer Schmelzeisen, Benedikt Christopher Spies, Felix Burkhardt, Marc Anton Fuessinger, René Marcel Rothweiler, Christian Gross, Stefan Schlager, Marc Christian Metzger

**Affiliations:** 1 Department of Oral and Maxillofacial Surgery, Clinic, Medical Center – University of Freiburg, Faculty of Medicine, University of Freiburg, Hugstetterstr. 55, 79106 Freiburg, Germany; 2 Key Scientist Modeling and Simulation, Fraunhofer Institute for Digital Medicine MEVIS, Bremen, Max-von-Laue-Str. 2, 28359 Bremen, Germany; 3 Department of Prosthetic Dentistry, Center for Dental Medicine, Medical Center –University of Freiburg, Faculty of Medicine, University of Freiburg, Hugstetterstr. 55, 79106 Freiburg, Germany

**Keywords:** Dental implant, Implant planning, Virtual surgery, Statistical shape model

## Abstract

**Purpose:**

The aim of the study was to assess the deviation between clinical implant axes (CIA) determined by a surgeon during preoperative planning and reconstructed tooth axes (RTA) of missing teeth which were automatically computed by a previously introduced anatomical SSM.

**Methods:**

For this purpose all available planning datasets of single-implant cases of our clinic, which were planned with coDiagnostix Version 9.9 between 2018 and 2021, were collected for retrospective investigation. Informed consent was obtained. First, the intraoral scans of implant patients were annotated and subsequently analyzed using the SSM. The RTA, computed by the SSM, was then projected into the preoperative planning dataset. The amount and direction of spatial deviation between RTA and CIA were then measured.

**Results:**

Thirty-five patients were implemented. The mean distance between the occlusal entry point of anterior and posterior implants and the RTA was 0.99 mm ± 0.78 mm and 1.19 mm ± 0.55, respectively. The mean angular deviation between the CIA of anterior and posterior implants and the RTA was 12.4° ± 3.85° and 5.27° ± 2.97° respectively. The deviations in anterior implant cases were systematic and could be corrected by computing a modified RTA (mRTA) with decreased deviations (0.99 mm ± 0.84 and 4.62° ± 1.95°). The safety distances of implants set along the (m)RTA to neighboring teeth were maintained in 30 of 35 cases.

**Conclusion:**

The RTA estimated by the SSM revealed to be a viable implant axis for most of the posterior implant cases. As there are natural differences between the anatomical tooth axis and a desirable implant axis, modifications were necessary to correct the deviations which occurred in anterior implant cases. However, the presented approach is not applicable for clinical use and always requires manual optimization by the planning surgeon.

## Introduction

Careful preoperative planning is inevitable to ensure the long-term success of dental implants [1]. While backward planning remains the planning method of choice, [1] the modalities by which it is carried out have changed fundamentally [2]: originally, surgeons planned implant placement based on two-dimensional X-rays, physical plaster casts and wax-ups produced manually by technicians [3]. In the following decades, sectional imaging evolved and became available for the vast majority of practitioners by the invention of Cone-Beam Computed Tomography (CBCT) [2]. In combination with the introduction of intraoral scanners and the development of computer-aided design and manufacturing, fully digital backward-planning workflows were described [4]. This did not only replace the costly fabrication of physical models, but also made dental implant surgery a more predictable procedure with favorable long-term results [5].

Despite the high amount of digitalization already present in most implant planning programs, the desired implant axis still has to be determined manually by a trained practitioner after fusion of a CBCT scan, an intraoral scan and a digital wax-up [6]. Therefore, the current workflow still appears too time-consuming and cumbersome for some clinicians [7]. Thus, technical planning vendors offer their services to take over the manual work and save the clinician the time-consuming assessment of the data-set. To completely forgo manual steps, and enhance the objectivity of the planning workflow, computer algorithms, which automatically determine a viable implant axis, would be desirable.

Statistical shape models (SSM) were introduced as a concept for virtual reconstruction of missing or defective parts of a known structure decades ago [8]. The original morphology of missing or defective parts of an anatomical structure can be calculated by an SSM based on a large number of anatomical training datasets [8]. In cranial and maxillofacial surgery, SSMs have been proposed for the virtual reconstruction of the skull, [9] the zygoma [10] or orbital floor defects [11]. Moreover, SSMs can be considered as a valuable tool that allows for fast and efficient reconstruction of dental anatomy [12–14]: In a previous study, we demonstrated that tooth axes of missing teeth can be reconstructed accurately based on the morphology of remaining tooth crowns using an anatomical SSM [13]. Furthermore, it has been suggested that the SSM-based reconstruction of root axes of missing teeth can provide useful information for virtual implant planning [13].

The aim of this study is to assess whether the previously introduced anatomical SSM can provide relevant information for implant planning despite the natural differences between an anatomical tooth axis and a clinically viable implant axis. Therefore, we compared anatomically reconstructed tooth axes (RTA) estimated by the SSM with clinical implant axes (CIA) determined by surgeons during preoperative planning. The hypothesis of this study is that there is a correlation between the two, which can supply useful information during virtual implant planning. The null hypothesis claims that there is no meaningful relation between the RTA and the CIA and, therefore, a clinical use of the RTA for implant purposes is not advisable.

## Materials and methods

In this retrospective monocentric study, dental implant cases of the Department of Oral and Maxillofacial Surgery of the Medical Center of the University of Freiburg, Germany, between 2018 and 2021, were reviewed. Due to the retrospective nature of this study, we implemented all available planning datasets. Therefore, no sample size calculation was conducted prior to data collection. All patients signed written consent for the scientific use of their data prior to study inclusion. The study was approved by the ethics committee of the University of Freiburg, Germany with the protocol (No. 21/1089).

### Collection of dental implant cases

Only datasets of dental implant cases with single-tooth gaps, planned with the Software coDiagnostiX, Version 9.9 (DentalWings, Montréal, Canada) between 2018 and 2021, were considered eligible for study inclusion.

The following inclusion criteria were defined:

- Legal age at the time of study implementation.
- Virtual surgical planning was finalized, and surgery was conducted following the virtual plan.
- Availability of a digital planning datasets containing a CBCT scan, an intraoral scan (IOS), a digital wax-up and a virtually placed implant.
- Single-tooth implant cases without any other teeth missing in region 16–26 or 36–46 (FDI-scheme)

#### Cone-Beam computed tomography

The CBCT scans were performed using the 3D Accuitomo 170 CBCT-scanner (Morita Corporation, Osaka, Japan). Datasets were exported as DICOM-files (Digital Imaging and Communications in Medicine) from the picture archiving and communication system (Agfa HealthCare IMPAX EE R20 XVII SU4, Mortsel, Belgium) and imported into coDiagnostiX, Version 9.9.

#### Intraoral scans (IOS)

**Fig. 1:**
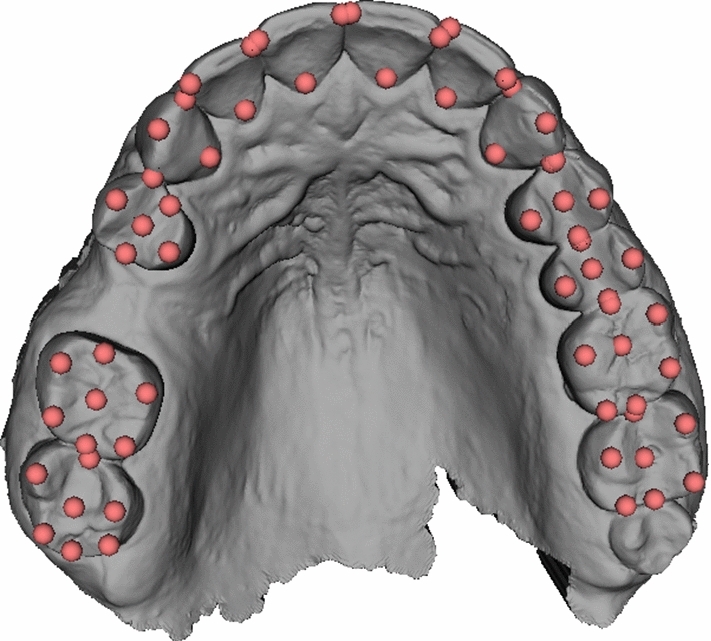
Annotated intraoral scan of patient #23

The intraoral scans were obtained using an intra-oral scanner (Trios 4, 3Shape, Copenhagen, Denmark). The scanning procedure was performed according to the manufacturer’s instructions. The mesh was exported as standard tessellation language (STL) file (see Fig. 1).

#### Virtual planning

The virtual planning of dental implant cases was conducted using coDiagnostiX, Version 9.9 (DentalWings, Canada). All implant cases were first planned by a resident physician and afterward approved by a senior physician. This approach is part of the local standard in our clinic, as it ensures dual control by two different surgeons. Residents were dentists in training for oral surgery and 2–4 years of experience. Seniors were certified oral surgeons with at least > 5 years of experience as oral surgeon. To guarantee restoratively driven backward planning, diagnostic wax-ups were used to estimate the best possible implant position. After thorough review of the virtual plan by a senior physician, an implant drill guide was created virtually and produced using an in-house 3D printer (Objet Eden260V™, Stratasys, Ltd.; Eden Prarie, Minn., USA). As virtual planning and surgery of the implemented cases was already finalized when data collection started, it was ensured that the planning physician was blinded from SSM-based results.

#### Anatomical landmarks

Anatomical landmarks were set on dental cusps, incisal edges and central fissures of remaining teeth as previously described [13] within the IOS using the open-source software 3D Slicer [15]. Landmarks in the region of the missing tooth were considered as missing values (see Fig. 1).

### Analysis of dental implant cases using the SSM

All tasks regarding SSM generation and application as well as the error assessment and statistical tests have been performed using the open source statistical platform R [16] and more specifically the packages Morpho, Rvcg [17], mesheR [18] and RvtkStatismo [19]. The anatomical SSM previously described by Brandenburg et al. was provided for this study [13].

#### Computation of reconstructed tooth axes (RTA) using the anatomical SSM

**Fig. 2:**
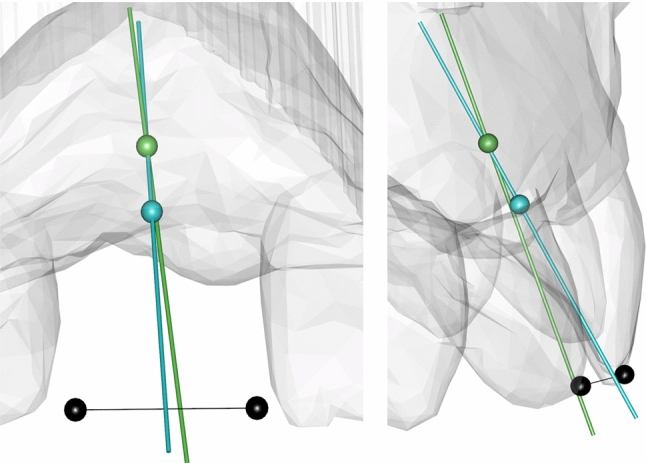
Dataset with missing tooth 11. The tooth-crown landmarks of the missing tooth were estimated by the SSM (black dots). A straight line passing through the occlusal and apical center point of the conventionally planned implant (green line) depicts the CIA with the corresponding occlusal entry point (green dot). The RTA (blue line) and the estimated cemento-enamel-junction line (CEJL, blue dot) deviate from the CIA

The set landmarks were used as only basis to compute the anatomical landmarks of the missing tooth crown and its associated anatomical root axis, further referred to as reconstructed tooth axis (RTA) (Fig. 2). For technical details of the SSM, see Brandenburg et al. [13].

#### Comparison of the clinical implant axis (CIA) and the reconstructed tooth axis (RTA)

The implant position virtually defined in the preoperative planning process was exported from coDiagnostiX, Version 9.9, as a cylindrical object with the dimensions and spatial alignment of the original implant (implant analogue; *.stl). Using the coordinates of the central axis of this cylinder, the clinical implant axis (CIA) was determined and compared with the reconstructed tooth axis (RTA) computed by the SSM (Fig. 2).

**Fig. 3:**
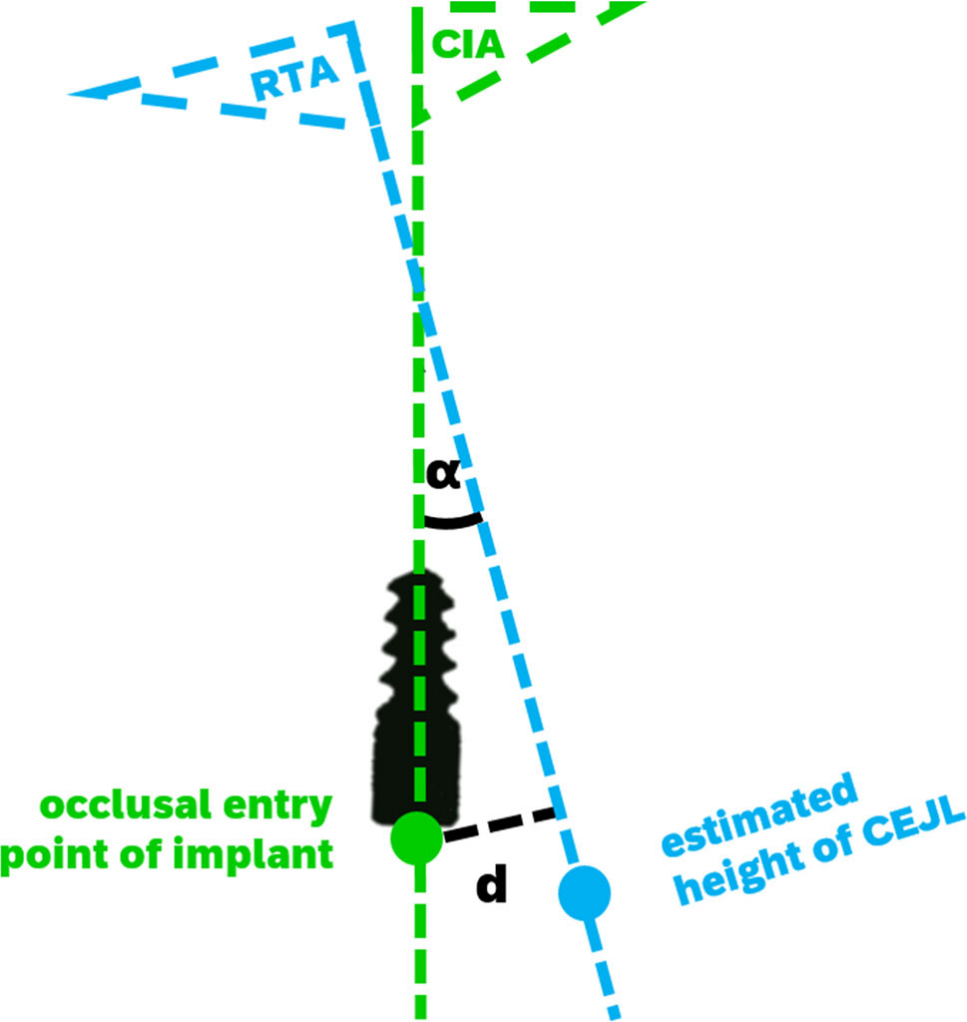
Scheme of measured errors. The distance d was measured as the shortest connection line between the occlusal entry point (green dot) of the implant (black) and the RTA (blue line). The angular deviation α was measured between the RTA and the CIA (green line)

To compare the RTA and the CIA, the deviations between these two were measured in distance and angle as previously reported [13] similar to the Consenus Report of the International Team for Implantology [20]:

The distance was measured between the occlusal center point of the clinically inserted implant and its shortest projection onto the RTA (Fig. 3).

The angle was determined by translating the vectors of the CIA and the RTA onto the origin and then computing the angle between those vectors (Fig. 3).

**Fig. 4:**
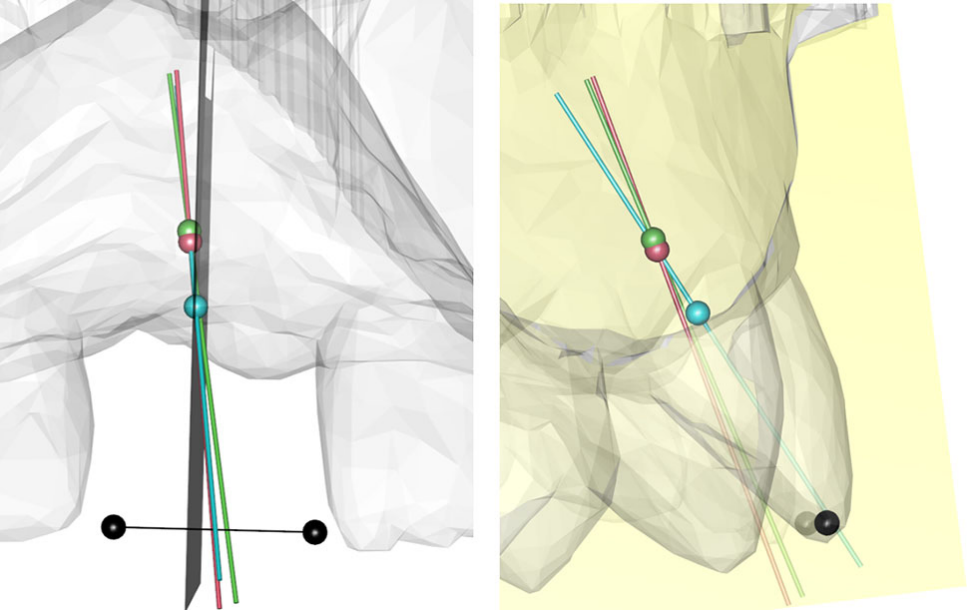
Determination of an oro-vestibular reference plane perpendicular to the incisal edges of the missing tooth (black dots) for further evaluation of the angular deviation. Left: The CIA (green line) with the occlusal entry point of the implant (green dot), the RTA (blue line) with the estimated height of the CEJL (blue dot) and the mRTA (red line) with the calculated pivotal-point (red dot) are depicted in a dataset with missing tooth 11. The reference plane is depicted as a gray rectangle, which is perpendicular to the estimated incisal edge (connection line of black dots) Right: Projection of the CIA (green), RTA (blue) and mRTA (red) onto the reference plane (yellow) for further analyses

For anterior teeth (canine to canine) we also computed a signed angle within a reference plane (yellow) to determine the direction of deviation. This reference plane was defined as a plane, which is perpendicular to the estimated incisal edge of the missing tooth (connection line between the black dots). The RTA and the CIA were projected onto this reference plane to determine the amount and direction of the angular deviation within the oro-vestibular plane to further analyze the existent deviation (Fig. 4).

### Computation of a modified reconstructed tooth axis (mRTA) and comparison with the clinical implant axis (CIA) of anterior implants

As the signed angular deviation of the RTA compared to the CIA of anterior implants (13–23 and 33–43, FDI-scheme) showed to be systematic (12.5° vestibular rotation in mean), we tried to revise this error by mathematical operations. For this purpose, the RTA was rotated within the previously defined reference plane (yellow, Fig. 4) by the average rotation-error measured in our study sample. To determine a viable pivot for this rotation, we calculated the mean deviation between the center of the occlusal entry point of the inserted implant and the estimated cemento-enamel-junction line (CEJL) given by the SSM. The pivot for the rotation was then placed apically (red dot, Fig. 4) to the estimated CEJL (blue dot Figs. 2, 3 and 4) along the RTA by the calculated amount. This yielded a modified RTA (mRTA, red line Fig. 4) which implements the mean angular deviation between the RTA and the CIA. The previously defined error metrics (distance and angular deviation) between the mRTA and the CIA were then recomputed.

#### Three-dimensional assessment of the mRTA within the CBCT dataset

**Fig. 5:**
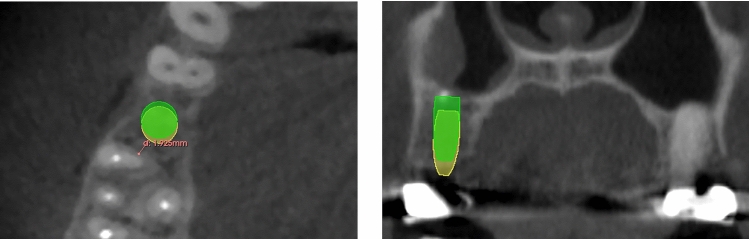
Example of a comparable accurate reconstruction result (patient #29) with an implant in region 15. The original implant is depicted in green. The implant placed along the mRTA is depicted in yellow

**Fig. 6:**
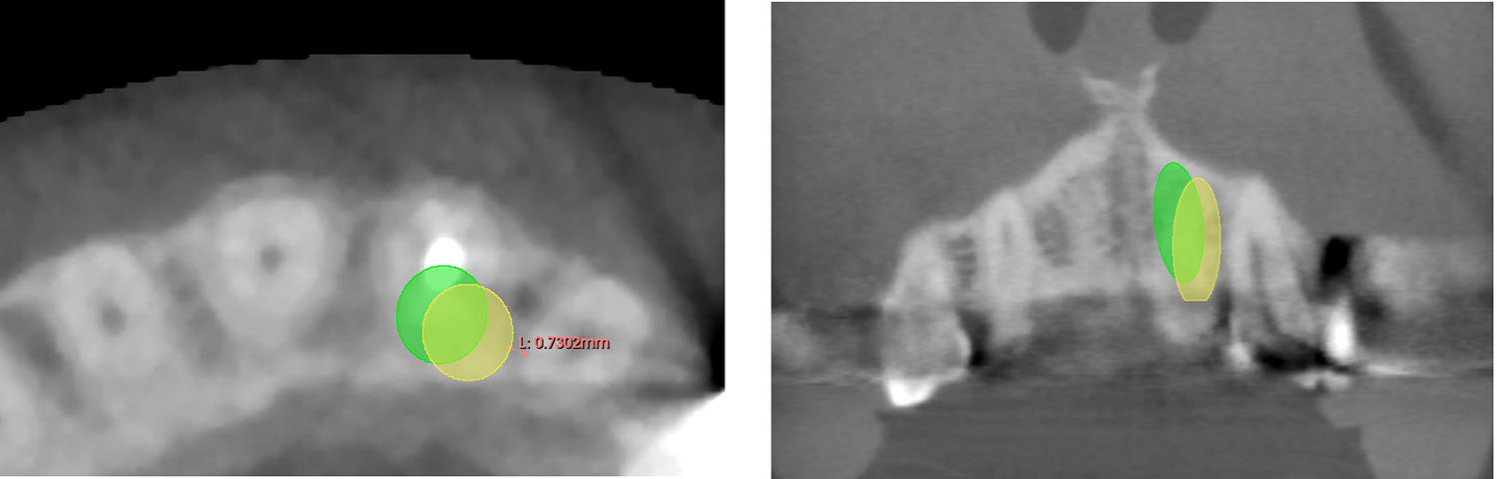
Example of a comparable poor reconstruction result (patient #34) with an implant in region 21. The original implant is depicted in green. The implant placed along the mRTA is depicted in yellow and appears to be shifted into a palatal-left direction leading to undercut safety distances to the neighboring tooth 23 (0.73 mm, see left image)

To assess whether the mRTA could be used as a valid orientation aid for dental implant positioning, it was checked whether implants set along the mRTA apply to the required safety distance of 1.5 mm to neighboring teeth as reported by Buser et al. [21]. For this purpose, the implant analogue of the original planning dataset was placed at the corrected occlusal entry point (red dot) along the mRTA (red line) (Fig. 4) and assessed within the CBCT dataset (Figs. 5 and 6).

## Results

### Study group

After application of the defined inclusion criteria, 35 Patients were included. Ten patients received a single implant in the anterior jaw and 25 patients received a single-implant in the posterior area (see Table 1).

**Table 1:** The deviations in angle and distance are shown in dependence of the investigated implant region

Patient number	Sex	Age	Implant region	Distance to RTA (mm)	Angle to RTA (°)	Distance to mRTA (mm)	Angle to mRTA (°)	Min. dist. to neighbor teeth (mm)	Perforation of cortical bone?
#1	m	47	32	− 6.32	6.68	0.23	5.9	1	No
#2	m	68	14	− 2.9	8.79			1.6	No
#3	m	27	11	− 3.1	19.5	2.8	7.55	2.1	Palatal
#4	m	60	26	− 4.39	6.43			4.6	No
#5	f	48	36	− 5.93	4.58			3.7	No
#6	f	80	46	− 1.59	6.68			4	No
#7	f	71	14	− 5.94	4.51			2.6	Vestibular
#8	m	29	22	− 2.86	14.73	0.81	2.55	1.6	No
#9	m	27	35	− 5.47	4.79			4.4	No
#10	f	51	23	− 4.77	10.7			0.7	Vestibular
#11	m	23	11	− 0.01	11.27	0.88	3.93	3.4	No
#12	m	34	36	− 6.65	7.31			5	No
#13	f	45	26	− 4.66	10.88			1.6	Vestibular
#14	m	73	16	− 1.09	4.88			4.1	No
#15	f	45	11	− 1.76	13.47	0.9	2.35	3.1	No
#16	f	70	15	− 5.02	2.36			2.1	No
#17	m	40	14	− 4.61	0.34			2.5	No
#18	m	46	11	− 3.43	10.14	0.01	3.54	2.7	No
#19	m	74	24	− 5.88	3.49			1.6	No
#20	m	50	15	− 3.26	13.3			3.1	No
#21	m	54	35	− 3.46	7.47			3	No
#22	m	37	16	− 0.1	2.31			2.6	No
#23	f	55	25	− 8.43	6.96			2.8	Vestibular
#24	f	51	15	− 2.97	3.67			2.3	No
#25	f	41	26	− 1.96	3.82			2.6	No
#26	f	69	36	− 2.04	4.78			3	No
#27	f	36	15	− 2.41	8.09			2.2	No
#28	f	64	24	− 4.76	2.7			1.6	No
#29	m	48	15	− 4.07	3.9			1.9	No
#30	m	64	22	− 4.06	17.31	0.49	5.89	1.6	No
#31	m	25	21	− 1.82	9.88	1.7	3.01	1.3	No
#32	f	59	24	− 5.08	3.72			1.3	No
#33	m	61	16	− 7.13	0.95			2.3	No
#34	f	49	21	− 1.5	10.39	1.17	6.81	0.7	No
#35	f	33	46	− 3.83	4.73			4.7	No

The recomputed error metrics of the mRTA are presented for the anterior region only, as they were not computed for posterior implant cases. The minimum distance to neighboring teeth and perforation of cortical bone was checked in the CBCT and protocolled in the two right-hand columns

### Comparison of the clinical implant axis (CIA) and the reconstructed tooth axis (RTA)

#### Anterior implant cases

The mean distance between the occlusal entry point of anterior implants and the RTA was 0.99 mm ± 0.78 mm (see Fig. 4). The mean angular deviation between the CIA of anterior implants and the RTA was 12.4° ± 3.85°.

#### Posterior implant cases

The mean distance deviation between the occlusal entry point of posterior implants and the RTA was 1.19 mm ± 0.55. The mean angular deviation between the CIA of posterior implants and the RTA was 5.27° ± 2.97°.

#### Comparison of errors in posterior and anterior implant cases

There was no significant difference for the distance deviations measured in anterior and posterior implant cases (*t*-test: *p* = 0.46).

The angular deviations of the CIA of anterior implants and the respective RTA were significantly larger than in posterior implant cases (*t*-test: *p* < 0.001).

The angular deviation between RTA and CIA in anterior implant cases revealed to be systematic with 12.5° palatal angulation in mean. The angular deviation of posterior implant cases were of random pattern.

### Computation of a modified reconstructed tooth axis (mRTA) for anterior implant cases

To create a modified RTA (mRTA) with decreased error metrics for anterior implant cases, the RTA was rotated 12.5° vestibular within the reference plane (yellow) (Fig. 4). The pivot of this rotation was placed 3 mm apical to the CEJL predicted by the anatomical SSM.

The mean distance deviation between the occlusal center point of anterior implants and the according mRTA was 0.99 mm ± 0.84. The mean angular deviation between the CIA of anterior implants and the corresponding mRTA was 4.62° ± 1.95°.

### Assessment of the (m)RTA within the CBCT dataset

Five of the implant analogues set along the mRTA undercut the safety distance of 1.5 mm to neighboring teeth when checked in the CBCT dataset. In two of the five cases even the conventionally positioned implant undercut the safety distance to a neighboring structure due to narrow anatomical conditions (see Table 1).

In both, the SSM-based positioned implant (along the RTA/mRTA) and the conventionally positioned implants (along the CIA) a perforation of the oral or vestibular cortical bone was present in five cases. Three of these five cases were identical.

Most implants positioned along the (m)RTA were placed occlusal to the clinically defined implant position (Figs. 5 and 6).

## Discussion

### The anatomical tooth axis as orientation aid in implant planning

Concerning the required distances to neighboring structures for proper implant positioning [21], the anatomical tooth axis appears to be a viable template for implant positioning: Especially in individuals with soundly formed dental arches, the dental roots are aligned regularly to each other and do not touch. As our anatomical SSM is built based on orthodontically treated patients, we assume that the RTA computed by the SSM implies a safety distance to neighboring dental roots. In the evaluated implant cases with single-tooth gaps, the SSM should therefore compute an RTA which is located in the middle of the two neighboring teeth and gives a maximum leeway space for implant surgery. Another useful aspect of the depiction of the tooth axis is that it estimates the original situation before tooth loss and may therefore reveal pathological alterations, which occurred due to edentulism in the implant region, such as bone loss. There is literature reporting that the critical vestibular bone dimensions around dental implants are different compared to the anatomically available bone. The presented workflow using an anatomical SSM could help to reveal areas which have a deficient bone supply and require bone augmentation prior to implant placement [22, 23].

The main disadvantage of the anatomical tooth axis is that it does not concern the anatomical peculiarities given in patients after tooth loss. Tooth migration and bone loss in the implant region, e.g. vestibular bone resorption, may lead to implant positions remarkably different to the anatomical root axis of the missing tooth. Due to esthetic considerations in the anterior area of the jaw, special requirements in implant placement add to this difficulty and may increase the differences between the anatomical tooth axis and a viable implant axis [21, 23].

### Comparison of the (m)RTA with the CIA

As the best possible implant position is clinically estimated based on a planning dataset consisting of a CBCT scan, an intraoral scan and a diagnostic wax-up, the desired implant position is influenced by the skills, experience and personal preferences of the planning surgeon [24]. As the SSM only uses anatomical landmarks of dental cusps to calculate an axis we did not expect the SSM to compute an (m)RTA which is identical to the CIA. Moreover, the determination of the modified RTA (mRTA) is ambiguous and subject to numerous errors as it is dependent of the precise location of the absent tooth’s incisal edge. However, we expected a correlation between the two which enables to gather useful information in implant-site assessment.

#### Posterior implant cases

The mean distance deviation between the occlusal entry point of posterior implants and the RTA was comparable to the reported inaccuracies of static computer-aided implant surgery (1.2 mm in distance and 3.5° in angle, ITI consensus report) [20] and showed to be insignificant (*t*-test: *p* = 0.58). The signed angles of posterior implant cases canceled out each other leading to a mean error near zero and were therefore considered to be random. When checked in the cross-sectional imaging, all posterior implants aligned along the RTA showed a minimum distance of 1.5 mm to neighboring teeth. This makes the RTA a clinically viable implant axis. The deviation in the apico-coronal direction developed most likely, because the occlusal entry point was derived from the anatomical CEJL.

#### Anterior implant cases

The mean distance deviation for implants in the anterior area was even lower than the reported inaccuracies of static computer-aided implant surgery [20]. The mean angular deviation between the CIA of anterior implants and the RTA was remarkably higher (12.4° ± 3.85°, *p* < 0.0001) than for posterior implant cases. Evaluating the signed angles within the reference plane (yellow) (Fig. 4), it could be shown that most of the observed deviations in anterior implant cases can be attributed to a vestibular tilt of the RTA of 12.5° in mean (Fig. 3). Therefore, the angular deviation of anterior implant cases was considered systematic. This tilt of 12.5° in mean most likely occurred, because the implants of the anterior region have to be set palatally to the former dental root to guarantee the maintenance of visible peri-implant gingival tissue [21]. The modification of the RTA (mRTA) yielded significantly lower deviations, which are comparable to the deviations observed in posterior implant cases. The systematic deviations detected in anterior implant cases indicate that there is a correlation between the anatomical alignment of a tooth and a dental implant, which is meant to replace its root. Therefore, when the observed deviations are concerned, the RTA could possibly be useful in determining implant position (see Figs. 5 and 6).

### Technical aspects of the proposed method and potentials for clinical use

The proposed method using an SSM infers the morphology of an absent anatomical structure (dental root) based on available anatomical information (landmarks on dental crowns). Once the training of the SSM is conducted, it captures the shape variability present in the training data. In contrast to conventional imaging techniques, the SSM does not visualize the actual anatomy of the patient, but an estimation based on the implemented training data. This carries the risk that the estimation is erroneous and infeasible for clinical use. The advantage is, that it can compute anatomical information without using radiation. The SSM used in the present study relies on a prototype, trained on less than 80 anatomical datasets. The performance of the SSM was already shown to be comparably high. By the implementation of additional training data, the accuracy may be increased further.

There is no doubt that cross-sectional imaging using CBCT is inevitable for the accurate determination of a desirable implant position. However, concerning the observed errors the presented method could be used to generate a first suggestion for virtual implant placement. As the deviations of the (m)RTA to the clinical implant position were shown to be small, the automated suggestion could be optimized easily by the planning surgeon to receive a clinically viable implant axis. This could accelerate the planning process and increase the efficacy of the virtual workflow. Moreover, there could be an advantage of the SSM-driven workflow by integrating the calculated CEJL to estimate the vertical position of the implant entry point. Based on the highest level of evidence, CBCT is required as a preoperative planning imaging modality prior to implant surgery following the position statement of the American Academy of Oral and Maxillofacial Radiology [25]. Therefore, the presented approach can only be seen as a starting point for further scientific research and can not be used clinically.

As there are inevitable differences between the RTA and the CIA, the development of an SSM, which implements clinically determined implant positions could be an alternative approach.

### Limitations of the study

The above mentioned differences between the dental root anatomy and implant positions, especially in the esthetic area, are one of the most important limitations of the proposed method. Moreover, the generalizability of the presented results is questionable, as only a small number of training datasets were implemented in the SSM. Likewise, the number of the evaluated implant cases is low and has to be enlarged to derive generalizability. Because of the limited experiences with the SSM-based method in dental implant planning the authors decided to first evaluate comparable simple implant cases with only one missing tooth and no additional pathological alterations. It has to be noted that complex implant cases may therefore not be viable for the reconstruction with the underlying anatomical SSM.

## Conclusion

In conclusion, it can be stated that the (m)RTA computed by the underlying SSM yielded viable implant axes for most of the posterior implant cases. The detected errors were similar to the inaccuracies present in static computer-aided implant surgery. For anterior implant cases a systematic error of 12.5° rotation into the palatal direction occurred when compared to the clinical implant axis. Therefore, in the esthetic area, modifications of the RTA were necessary to correct for the systematic deviations. The modified RTA (mRTA) showed significantly lower deviations. Despite the promising results, it has to be noted that the automatically generated implant axes could have been used as a first suggestion only, with the need for manual optimization. Even if this could yield an acceleration of the planning process, the reliability of the proposed method still has to be validated further before its clinical application. Future efforts should aim to increase the accuracy of the SSM. Moreover, instead of dental roots, it could be more viable to use postoperative scans of dental implants as training data.
